# Derma-Hc, a New Developed Herbal Formula, Ameliorates Cutaneous Lichenification in Atopic Dermatitis

**DOI:** 10.3390/ijms22052359

**Published:** 2021-02-26

**Authors:** Yeon Kyung Nam, Mi Hye Kim, In Jin Ha, Woong Mo Yang

**Affiliations:** 1 Department of Convergence Korean Medical Science, College of Korean Medicine, Kyung Hee University, Seoul 02447, Korea; nyk7705@khu.ac.kr (Y.K.N.); kimmihye526@khu.ac.kr (M.H.K.); 2 Korean Medicine Clinical Trial Center, Kyung Hee University Korean Medicine Hospital, Kyung Hee University, Seoul 02454, Korea; ijha0@naver.com

**Keywords:** Derma-Hc, atopic dermatitis, lichenification, dry skin, keratinocyte

## Abstract

Atopic dermatitis (AD) is a chronic cutaneous disorder that is characterized by severe eczematous inflammation, swelling, and lichenification. Activation of T helper (Th)-22 cells by allergens leads to epidermal hyperplasia with hyperkeratosis at the chronic phase of AD. Derma-Hc is composed of five natural herbs with anti-AD effects, such as *Astragalus membranaceus* BUNGE, *Schizonepeta tenuifolia* Briq., *Cryptotympana pustulata* Fabr., *Angelica sinensis* Diels, *Arctium lappa* L. In this study, the ameliorative effect of Derma-Hc on cutaneous lichenification in 2,4-dinitrochlorobenzne (DNCB)-induced AD was investigated. The dorsal skin of mice was sensitized with DNCB to induce AD-like skin lesions. The dermatitis score and frequency of scratching were evaluated. Thickness of epidermis and dermis was measured by staining with H&E. In addition, infiltration of the mast cell was observed by staining with toluidine blue. Then, desmosomal cadherin, DSC1 was examined by immunofluorescence. Pathological mechanisms involved in lichenification were analyzed in AD-like skin lesions and TNF-α + IFN-γ-treated with human keratinocytes including keratinocyte differentiation genes and JAK1-STAT3 signaling pathway with IL-22 by RT-PCR and western blotting. Topical treatment of Derma-Hc improved AD-like symptoms such as dryness, edema and lichenefication and decreased the number of scratches. Histopathological analysis demonstrated that Derma-Hc significantly inhibited epidermal hyperplasia, hyperkeratosis, and mast cells infiltration. In addition, the level of DSC1 was highly expressed in the epidermis by Derma-Hc. Moreover, mRNA expression level of FLG, an epidermal differentiation complex gene, was recovered by Derma-Hc treatment. KLK5 and KLK7 were markedly reduced to normalize keratinocyte differentiation in dorsal skin tissues and human keratinocytes. On the other hand, Derma-Hc restored expression level of SPINK5. In addition, Derma-Hc inhibited IL-22 via the blockade of JAK1-STAT3 signal pathway. Taken together, Derma-Hc, a natural herbal formula, regulated keratinocyte differentiation and inhibited epidermal hyperplasia with hyperkeratosis. Therefore, Derma-Hc could be a promising candidate for treating chronic AD through modulating signaling of IL-22-associated skin lichenification.

## 1. Introduction

Atopic dermatitis (AD) is multifactorial inflammatory skin disease characterized by pruritus, edema, eczema, xerosis cutis, and lichenification [1,2]. The cause of AD has not been fully understood, however, genetic factors, hygiene theory, allergen and abnormalities in the immune system are highly involved in pathogenesis of AD [3]. The incidence of AD has been increasing, affecting up to 20% of children and 10% of adult in industrialized countries [4]. The psychological and economic burdens lead to deterioration of the quality of AD patient’s life [5]. Exacerbation of itching-scratching cycle result in lichenification [6]. Atopic skin becomes leathery, scaly, and thickened by continuously scratching or rubbing skin lesions due to chronic irritation [7]. Therefore, molecular mechanisms in lichenified skin lesions should be elucidated to manage chronic AD.

The pathomechanism of AD is featured by dysregulation of immune responses that are related to T-cell dominant inflammation and allergen hypersensitivity [8]. Especially, T cells, such as T helper (Th)1, Th2, Th17, and Th22 cells are critical mediators which initiate progression of AD in response to an allergen [9]. Upon sensitization to an allergen, IgE produced by B cell bound high affinity-IgE receptors and increased mast cell degranulation to release histamine and cytokines [10,11]. Th2/Th22 cell polarization produces cytokines, interleukin (IL)-4, IL-13, IL-31, and IL-22 in the acute phase of AD. On the other hand, Th1, Th17, and Th22 cells are activated, and associated cytokines were secreted in the chronic phase [12]. In addition, Th22 cell is highly contributed to chronic pruritus, dermatitis and skin barrier impairment by amplifying inflammation on keratinocytes [8]. IL-22 derived from Th22 cells specifically binds the heterodimeric receptor and activates janus kinase 1 (JAK1), signal transducer and activator of transcription 3 (STAT3) leading to the inhibition of terminal differentiation of keratinocytes and induces epidermal hyperplasia [13]. Stimulation of IL-22 in keratinocytes downregulates the expression of filaggrin (FLG), which further worsens activity between protease and protease inhibitor such as kallikrein-related peptidase 5 (KLK5), kallikrein-related peptidase 7 (KLK7) and serine protease inhibitor Kazal-type 5 (SPINK5) [14]. Then, KLK5 and KLK7 induced proteolysis of desmocollin 1 (DSC-1), one of corneodesmosonal components, which is involved in hyperkeratosis [15]. In this study, IL-22/Th22 cells-mediated epidermal thickening and the underlying mechanism as a major route of lichenification observed in chronic AD phase was investigated in vivo and in vitro models.

Therefore, therapeutic management of AD has been practiced through targeting T cell pathways that block specific molecules involved in itching and inflammatory cascade [16,17]. Treatment with the corticosteroids and monoclonal antibodies classified into immunosuppressive drugs have been prescribed to reduce itching and skin inflammation for AD patients [18,19]. However, long-term use of corticosteroids induces side effects including local skin atrophy, thinning skin, osteoporosis, and thrombosis [20]. Although calcineurin inhibitors, alternatives to corticosteroids, exert anti-inflammatory effects without local skin atrophy, the risk of malignancy, gingival hyperplasia and facial flushing is increased by prolonged usage [21]. Therefore, complementary therapies with natural herbs might be pivotal strategies for the treatment of AD.

Derma-Hc consists of five herbs including *Astragalus membranaceus* BUNGE (AM), *Schizonepeta tenuifolia* Briq. (ST), *Cryptotympana pustulata* Fabr. (CP), *Angelica sinensis* (Oliv.) Diels (AS), and *Arctium lappa* L. (AL), which have been traditionally used for treating skin disorders in Korea. In addition, the above five medicinal herbs have efficacy in maintaining coordination and balance by inhibiting blood heat and blood stasis in AD-like skin in terms of traditional Korean medicine [22]. Previous study has reported that Derma-H, mixture of AM and ST exerted anti-inflammatory and anti-pruritus effects by inhibiting the production of cytokines on AD skin [23]. In particular, AM reduced epidermal hyperplasia and hyperkeratosis in AD skin lesions [24]. ST topical treatment suppressed inflammatory factors in the serum of AD [25]. Furthermore, CP, AS, and AL were determined addition to Derma-H based on our research. CP has been known for treatment of allergic skin diseases according to analyzed prescription database [26]. In addition, AS improved skin inflammation by downregulating secretion of cytokines [27]. AL has anti-allergic activity on IgE-mediated hypersensitivity [28]. Therefore, Derma-Hc consisting of five medicinal ingredients might be expected to verify the phytotherapeutic potential of AD. We put emphasis on the IL-22-associated mechanism including characterized keratinocyte differentiation markers as well as AD-like phenotype.

## 2. Results

### 2.1. Effect of Derma-Hc on Morphological Phenotype and Dermatitis Score

**Figure 1:**
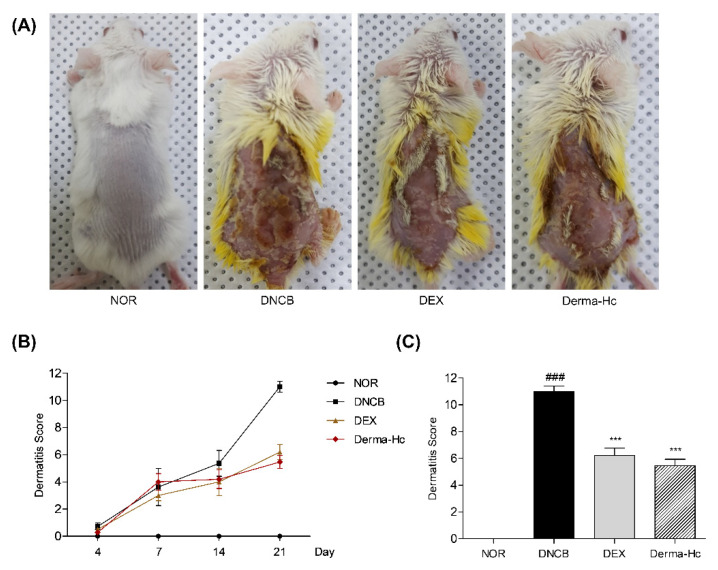
Derma-Hc attenuates the development of atopic dermatitis in DNCB-induced mice. NOR, Normal saline-treated group; DNCB, 2,4-dinitrochlorobenzene (DNCB)-sensitized group; DEX, DNCB-sensitized and dexamethasone-treated group. (**A**) Photographs of the dorsal skin lesions from individual groups of 5-week-old male BALB/c mice (*n* = 5) at the end of the experiment (Day 21). (**B**,**C**) The dermatitis score was measured on the basis of atopic dermatitis-like symptoms including erythema/hemorrhage, scarring/dryness, edema, and excoriation/erosion. The data are presented as mean ± standard error of the mean. ^###^ *p* < 0.001 compared to NOR group. *** *p* < 0.001 compared to individual groups.

During the experiment, 20 mg/mL of Derma-Hc was topically administered. Derma-Hc was identified as one of the seven chemical components such as calycosin 7-O-β-glucoside, nodakenin, hesperidin, luteolin, formonoetin, astragaloside IV, and astragaloside I using UPLC-ESI-QTOF MS/MS. To investigate the therapeutic effects of Derma-Hc on skin lesions in DNCB-induced AD mice models, the dermatitis scores were measured on days 4, 7, 14, and 21. In addition, the morphological change was observed by taking a photograph just before the sacrifice. DNCB group exhibited severe symptoms with erythema/hemorrhage, scarring/dryness, edema, and excoriation/erosion. DNCB treatment presented total dermatitis scores on day 21 as 11 ± 0.82. The final dermatitis score measured with AD symptoms were improved by 43.64% (6.2 ± 0.56) and 50.3% (5.46 ± 0.47) when topically treated with DEX and Derma-Hc compared to DNCB (Figure 1).

### 2.2. Effect of Derma-Hc on Scratching Behavior

**Figure 2:**
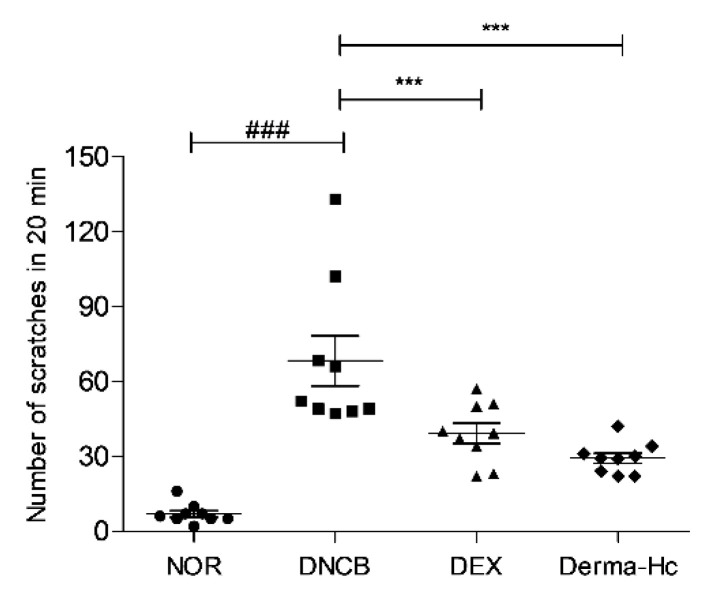
Derma-Hc improves the frequency of scratching behavior in DNCB-induced mice. NOR, Normal saline-treated group; DNCB, 2,4-dinitrochlorobenzene (DNCB)-sensitized group; DEX, DNCB-sensitized and dexamethasone-treated group. The number of scratching was counted for 20 min through watching recorded video. The data are presented as mean ± standard error of the mean. ^###^ *p* < 0.001 compared to NOR group. *** *p* < 0.001 compared to individual groups.

The number of scratching was counted because itching is the hallmark of AD. DNCB treatment increased the scratching behaviors about 9.8-folds (68.25 ± 10) compared to the NOR group. Administration of DEX and Derma-Hc reduced the frequency of scratching behavior reduced by 42.49% (39.25 ± 4.02) and 57.14% (29.25 ± 2.11), respectively (Figure 2).

### 2.3. Effect of Derma-Hc on Skin Thickness

**Figure 3:**
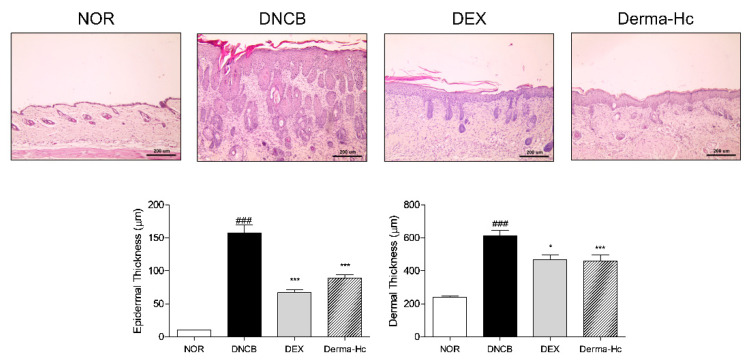
Derma-Hc ameliorates histological features presented in atopic dermatitis-like lesions of DNCB-induced mice. NOR, Normal saline-treated group; DNCB, 2,4-dinitrochlorobenzene (DNCB)-sensitized group; DEX, DNCB-sensitized and dexamethasone-treated group. Representative images of dorsal skin tissues were stained with H&E. Blue, cell nuclei; Pink, extracellular matrix and cytoplasm. The thickness of epidermis and dermis was measured after tissue sections were observed under a microscope (magnification 100×, scale bar 200 μm). The data are presented as mean ± standard error of the mean. ^###^ *p* < 0.001 compared to the NOR group. * *p* < 0.05 and *** *p* < 0.001 compared to individual groups.

Histological change of dorsal skin tissues was assessed by staining with H&E. Microscopic analysis indicated that dorsal skin lesions exhibit epidermal thickness, epidermal hyperplasia and hyperkeratosis leading to skin lichenification. The epidermal thickness in the DNCB group was increased about 15.6-folds (157.6 ± 12.12 μm) compared to the NOR group. Topical administration of DEX and Derma-Hc was significantly decreased by 57.54% (66.91 ± 4.6 μm) and 43.69% (88.74 ± 5.34 μm) compared with the DNCB group. Moreover, dermis thickened to 2.6-folds (612.9 ± 31.39 μm) by DNCB treatment due to the infiltration of inflammatory cells. DEX and Derma-Hc application to dorsal skin showed a significant decrease of about 23.67% (467.8 ± 26.97 μm) and 24.87% (460.5 ± 24.87 μm) on the thickness of dermis in Figure 3.

### 2.4. Effect of Derma-Hc on Mast Cell Infiltration

**Figure 4:**
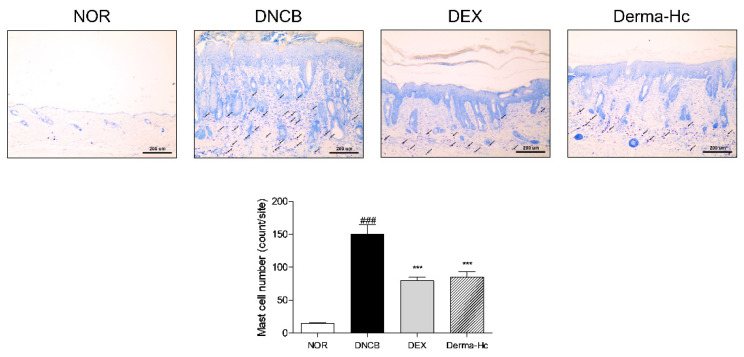
Derma-Hc reduces infiltration of mast cells in DNCB-induced mice. NOR, Normal saline-treated group; DNCB, 2,4-dinitrochlorobenzene (DNCB)-sensitized group; DEX, DNCB-sensitized and dexamethasone-treated group. Mast cells in dermis were stained with toluidine blue indicated as black arrow and presented as purple. Scale bar is 200 μm. The number of mast cells in five sites randomly designated were counted by Image J program. The data are presented as mean ± standard error of the mean. ^###^ *p* < 0.001 compared to NOR group. *** *p* < 0.001 compared to individual groups.

Toluidine blue staining was performed to observe a grade of mast cell production under the optical microscope. As illustrated in Figure 4, the number of mast cells in the dermis layer increased about 10.2-folds (150.3 ± 14.27) by DNCB treatment. On the other hand, infiltrated mast cells were attenuated approximately 47.15% (79.44 ± 5.57) and 43.3% (85.22 ± 8.21) when applied to DEX and Derma-Hc.

### 2.5. Effect of Derma-Hc on Expression Level of DSC1

**Figure 5:**
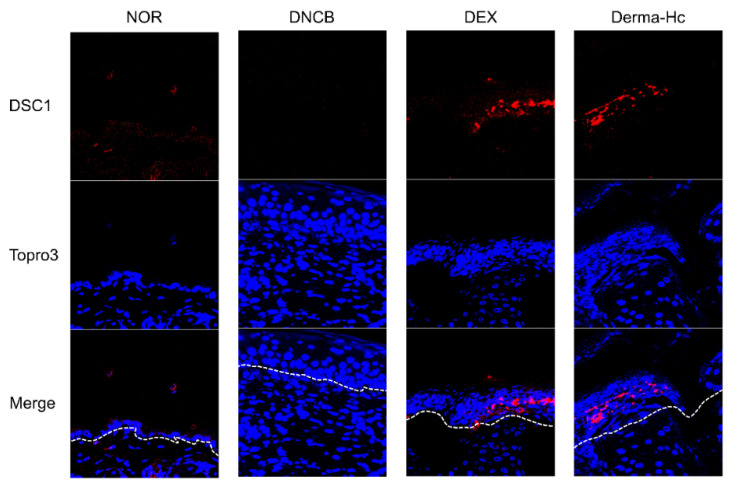
Derma-Hc up-regulates the expression level of DSC1 in DNCB-induced mice. NOR, Normal saline-treated group; DNCB, 2,4-dinitrochlorobenzene (DNCB)-sensitized group; DEX, DNCB-sensitized and dexamethasone-treated group. The pattern of DSC1 was detected by immunofluorescence analysis. Nuclei (blue) was stained with TO-PRO-3. DSC1 (red) was expressed in the layer of stratum granulosum and stratum spinosum in epidermis.

We assessed the expression of DSC1 in AD-like skin lesions when treated with Derma-Hc. Immunofluorescence staining illustrated that DSC1 in upper epidermis was drastically reduced in DNCB group compared to NOR. However, DEX and Derma-Hc treatment extensively increased the expression level of DSC1 in stratum granulosum and stratum spinosum of epidermis (Figure 5).

### 2.6. Effect of Derma-Hc on Expression Level of FLG

**Figure 6:**
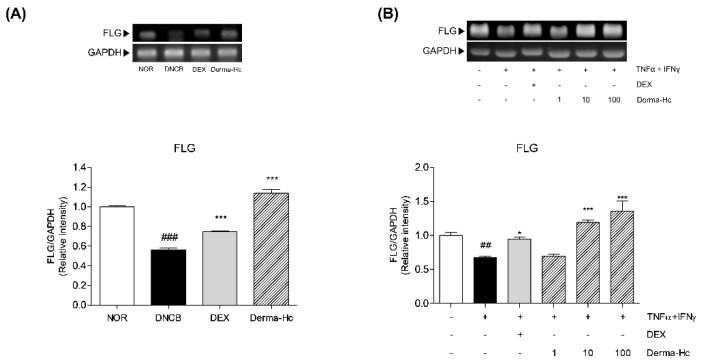
Derma-Hc recovers the expression level of FLG in (**A**) DNCB-induced mice and (**B**) TNF-α+IFN-γ-treated human keratinocytes. NOR, Normal saline-treated group; DNCB, 2,4-dinitrochlorobenzene (DNCB)-sensitized group; DEX, DNCB-sensitized and dexamethasone-treated group. The mRNA level of FLG was analyzed by RT-PCR. Three repeats of an experiment were conducted. The data are presented as mean ± standard error of the mean. ^##^ *p* < 0.01 and ^###^ *p* < 0.001 compared to NOR group. * *p* < 0.05 and *** *p* < 0.001 compared to individual groups. FLG, filaggrin; GAPDH, Glyceraldehyde 3-phosphate dehydrogenase.

To determine the effect of Derma-Hc administration on the FLG and EDC markers in AD skins, mRNA levels were measured by RT-PCR. Expression level of FLG was lowered by about 43.66% (0.56 ± 0.02) in DNCB application than that in vehicle control. Topical treatment of Derma-Hc increased the FLG level about 2-folds (1.14 ± 0.04) (Figure 6A).

Likewise, the expression mRNA level of FLG in TI-treated HaCaT cells was analyzed. TI treatment decreased the mRNA level of FLG by 32.57% (0.67 ± 0.02). Derma-Hc 10 and 100 μg/mL treatment in TI-treated cells enhanced the expressions of FLG by 2.5-folds (1.19 ± 0.04) and 2.9-folds (1.36 ± 0.14) (Figure 6B).

### 2.7. Effect of Derma-Hc on Expression Levels of Keratinocyte Differentiation-Related Factors

**Figure 7:**
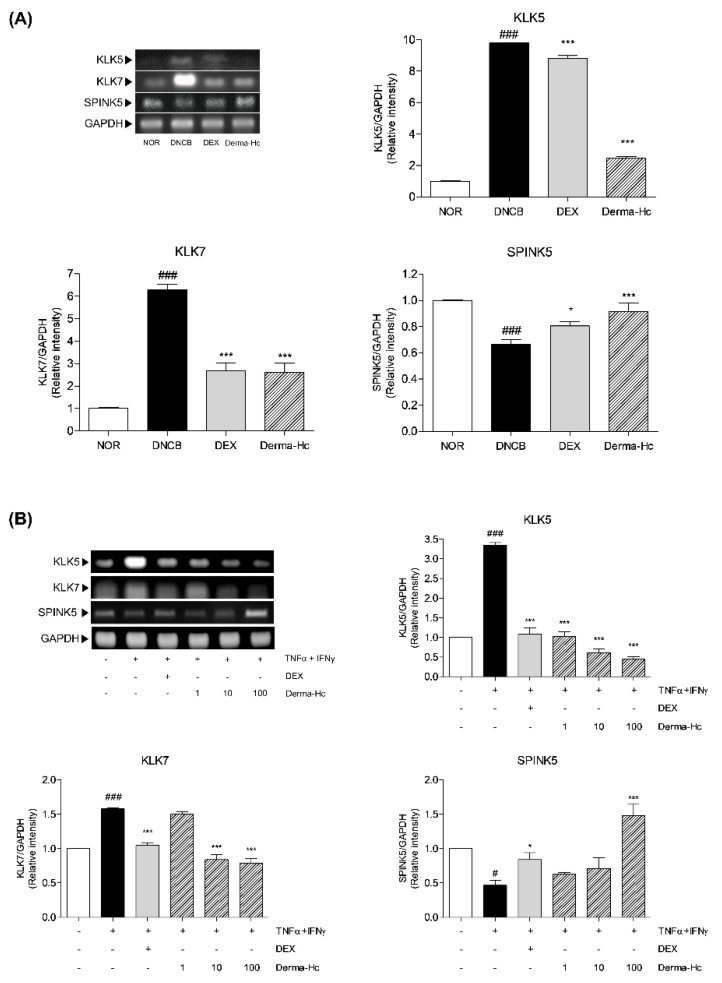
Derma-Hc regulates the expression levels of keratinocyte differentiation-related factors in (**A**) DNCB-induced mice and (**B**) TNF-α+IFN-γ-treated human keratinocytes. NOR, Normal saline-treated group; DNCB, 2,4-dinitrochlorobenzene (DNCB)-sensitized group; DEX, DNCB-sensitized and dexamethasone-treated group. The mRNA levels of kallikrein-related peptidase 5 (KLK5), kallikrein-related peptidase 7 (KLK7) and serine protease inhibitor Kazal-type 5 (SPINK5), were analyzed by RT-PCR. Three repeats of an experiment were conducted. The data are presented as mean ± standard error of the mean. *^#^ p* < 0.05 and ^###^ *p* < 0.001 compared to NOR group. * *p* < 0.05 and *** *p* < 0.001 compared to individual groups.

mRNA levels of skin barrier-related factors including KLK5, KLK7 and SPINK5 were measured by RT-PCR to verify the effect of Derma-Hc administration in dorsal skins. Expressions of KLK5 and KLK7 were higher in 9.8-folds (9.8 ± 0.03) and 6.3-folds (6.28 ± 0.24) in the dorsal skin of DNCB-treated mice compared to the control group. On the contrary, the expression level of SPINK5 was decreased by 33.47% (0.67 ± 0.04) in the DNCB-treated group compared to NOR. Derma-Hc downregulated expressions of KLK5 and KLK7 by 74.83% (2.47 ± 0.12) and 58.54% (2.61 ± 0.41), respectively. In addition, the mRNA level of SPINK5 is upregulated by about 1.4-folds (0.91 ± 0.07) by the Derma-Hc treatment (Figure 7A).

Moreover, the expression of mRNA levels of KLK5, KLK7, and SPINK5 were analyzed in TI-treated HaCaT cells. Expression levels of KLK5 and KLK7 were increased by 3.3-folds (3.34 ± 0.07) and 1.6-folds (1.58 ± 0.001) by TI treatment. In contrast, TI treatment decreased the mRNA level of SPINK5 by 53.21% (0.47 ± 0.08). However, RT-PCR analysis showed that the expression level of KLK5 was dose-dependently lowered by 69.16% (1.03 ± 0.1), 81.65% (0.61 ± 0.1) and 86.8% (0.44 ± 0.07) in Derma-Hc 1, 10 and 100 μg/mL treatment. In addition, treatment of Derma-Hc 10 and 100 μg/mL significantly decreased the level of KLK7 by about 46.73% (0.84 ± 0.07) and 49.89% (0.79 ± 0.07). The expression level of SPINK5 remarkably recovered about 3.2-folds (1.48 ± 0.17) using the Derma-Hc 100 μg/mL treatment. (Figure 7B).

### 2.8. Effect of Derma-Hc on Expression Levels of JAK1-STAT3 Signal Pathway

**Figure 8:**
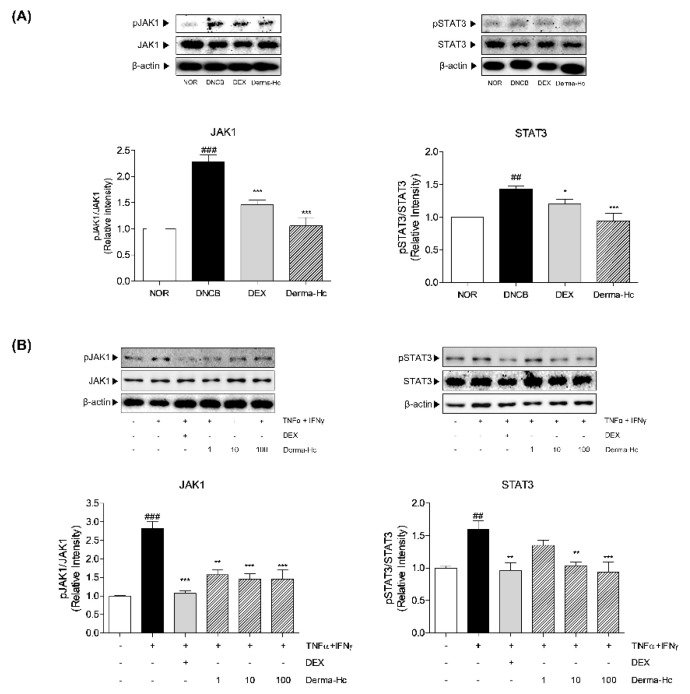
Derma-Hc decreases the expression levels of janus kinase 1 (JAK1)-signal transducer and activator of transcription 3 (STAT3) signal pathway in (**A**) DNCB-induced mice and (**B**) TNF-α+IFN-γ-treated human keratinocytes. The protein levels of phosphorylated janus kinase 1 (pJAK1) and phosphorylated signal transducer and activator of transcription 3 (pSTAT3) were analyzed by western blot assay. Three repeats of an experiment were conducted. The data are presented as mean ± standard error of the mean. ^##^ *p* < 0.01 and ^###^ *p* < 0.001 compared to NOR group. * *p* < 0.05, ** *p* < 0.01 and *** *p* < 0.001 compared to individual groups.

The effect of Derma-Hc on the JAK1-STAT3 pathway, known as downstream of IL-22 signaling was analyzed in DNCB-induced mice using western blot assay. The expression of JAK1 and STAT3 was significantly phosphorylated by about 2-folds (2.03 ± 0.14) and 1.4-folds (1.43 ± 0.03) in DNCB-treated mice compared to vehicle-treated mice. However, Topical application of Derma-Hc downregulated by 39.65% (1.23 ± 0.21) and 34.2% (0.94 ± 0.12) on JAK1-STAT3 phosphorylation compared with DNCB treatment (Figure 8A).

Similarly, dose-dependent treatment of Derma-Hc was induced in TI-treated HaCaT cells. Expose to TNF-α and IFN-γ in human keratinocytes induced the increase of expression of phosphorylated JAK1 and STAT3 about 2.8-folds (2.82 ± 0.19) and 1.6-folds (1.59 ± 0.14) compared with untreated control. Reduction in expression of pJAK1 presented to be dose-dependent manner about 44.39% (1.57 ± 0.14), 48.32% (1.46 ± 0.14), and 48.39% (1.46 ± 0.26) by Derma-Hc 1, 10 and 100 μg/mL treatment. In addition, Derma-Hc 10 and 100 μg/mL treatment inhibited the expression of the protein level of pSTAT3 by 34.86% (1.03 ± 0.06) and 40.9% (0.94 ± 0.15) compared to HaCaT cells treated with TI (Figure 8B).

### 2.9. Effect of Derma-Hc on Expression Level of IL-22

**Figure 9:**
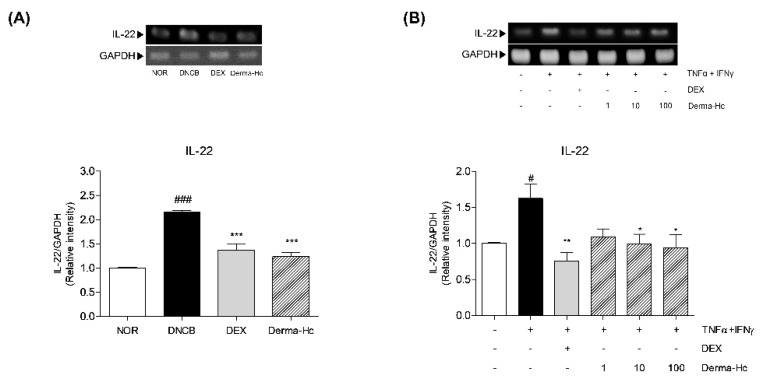
Derma-Hc down-regulates the expression level of IL-22 in (**A**) DNCB-induced mice and (**B**) TNF-α+IFN-γ-treated human keratinocytes. The mRNA level of IL-22 was measured by RT-PCR. Three repeats of an experiment were conducted. The data are presented as mean ± standard error of the mean. ^#^ *p* < 0.05 and ^###^ *p* < 0.001 compared to NOR group. * *p* < 0.05, ** *p* < 0.01 and *** *p* < 0.001 compared to individual groups.

To confirm the effect of Derma-Hc on the expression of IL-22 elicited from Th22 cell, RT-PCR was conducted in dorsal tissues of each mouse. The relative mRNA level of IL-22 was increased by 2.1-folds (2.15 ± 0.04) in DNCB-induced mice compared to the NOR group. Topical DEX and Derma-Hc treatment reduced the expression of IL-22 by 36.22% (1.37 ± 0.13) and 42.49% (1.24 ± 0.09) in comparison with DNCB treatment (Figure 9A).

In addition, the expression of IL-22 production was measured in TI-stimulated HaCaT cells. As shown in Figure 9B, expression level of IL-22 was increased by 1.6-folds (1.62 ± 0.19) in TI-treated cells compared to non-treated cells. By contrast, Derma-Hc 10 and 100 μg/mL treatment decreased the level of IL-22 about 38.89% (0.99 ± 0.13) and 42.08% (0.94 ± 0.18) compared to TI-treated cells (Figure 9B).

## 3. Discussion

AD is the most common chronic inflammatory cutaneous disease related with skin hyper-reactivity to pathogenic triggers [29]. AD is caused by complex immunological pathways including skin barrier dysfunction, genetic susceptibility and dysregulation of the immune system [30]. Clinically, consequent symptoms such as pruritus, facial or extensor eruptions, relapsing dermatitis and lichenification were described as a diagnostic standard of AD by Hanifin and Rajika [31]. Alternative treatments from natural herbs have been suggested to prevent and improve AD in recent studies [32]. In our previous study, Derma-H as an effective topical ointment reduced pruritus and inflammation by inhibiting NGF-TrKA signal pathway in DNCB-induced AD skin lesions [23]. A polysaccharide from *Cryptotympana pustulata* of Derma-Hc has been used for retaining the moisture in skin [33]. In addition, another study demonstrated that *Angelica sinensis* has an effect on preventing abnormal epidermal proliferation [27]. In addition, *Arctium lappa* has been known for improving quality and texture of skin by circulating blood into the skin surface [34]. For that reason, Derma-Hc including Derma-H with three herbs was assumed to improve chronic AD symptoms such as hyperkeratosis and lichenification, resulting from severe itching.

The key signs of AD including erythema/hemorrhage, scarring/dryness, edema, and excoriation/erosion were evaluated by proportionally assigned dorsal skin to assess the accurate development of AD-like skin lesions [35]. The sum of individual scores was increased in the negative control group. The administration of Derma-Hc remarkably improved signs of AD by approximately half. These results demonstrated that Derma-Hc suppressed the development of AD by inhibiting the symptomatic intensity of AD. In addition, itching, a typical feature in AD, is an uncontrollable sensation which provokes scratching [36]. A constant ‘itch-scratch cycle’ causes more inflammation and rash, which further disrupts the cutaneous barrier [37]. In this study, topical treatment of Derma-Hc reduced scratching movement resulting in ameliorating pruritic symptoms.

Excessive scratching and rubbing also induce epidermal hyperplasia in lichenified AD skin [38,39]. Epidermal hyperplasia, called acanthosis, is described as excessive proliferation of keratinocytes in the chronic phase of AD [40]. Hyperkeratosis associated with hyperplasia is dominant in the stratum corneum, which is the outermost layer of the epidermis [41]. Therefore, thickness of epidermis and dermis was measured to evaluate the effect of Derma-Hc on the extent of epidermal hyperplasia and hyperkeratosis by staining with H&E. Moreover, mast cells play an important role in the immediate hypersensitivity to allergic diseases [42]. It has been known that the activation of mast cells increased the number of mast cells [43]. We further performed toluidine blue staining to confirm the effect of Derma-Hc on the infiltration of mast cells. Our data provided that Derma-Hc has an inhibitory effect on the release of mast cells and epidermal and dermal thickening.

During the process of epidermal differentiation, expression of desmosomal cadherin, also called desmocollin, has an effect on the cutaneous morphological phenotypes [44]. One of the components of intracellular desmosome junctional protein, DSC1 is expressed between granular and spinous layers mediating the cohesion of HaCaT cells [45]. However, according to activation of protease such as KLK5 and KLK7, the junctional structures are degraded in cell-cell adhesion [15]. Consequently, loss of keratinocyte adhesion was correlated with hyperkeratosis in the uppermost corneocytes [46]. Therefore, we hypothesized that the expression of DSC1 affects AD-like skin lesions by activating KLK-associated peptidases. In this study, the expression level of DSC1 is downregulated in the epidermis of DNCB-induced skin, whereas, distribution of DSC1 is increased by Derma-Hc treatment. This result confirmed that Derma-Hc restored the expression of DSC1 by inhibiting induction of weakened adhesion.

In regard to skin barrier function, the epidermal barrier is composed of highly flattened and differentiated keratinocytes, thereby protecting against pathogens and avoiding loss of moisture from skin [47]. *FLG* is a critical gene for the structure of the epidermis which is located in epidermal differentiation complex (EDC) [48]. FLG degraded by proteolysis maintains epidermal hydration, the normal pH gradient of epidermis and skin barrier function [49]. FLG deficiency or null mutation leads to epidermal dysfunction, resulting in vulnerability and sensitization to allergen and epidermal hyperplasia [30,50]. In this study, the mRNA level of FLG was decreased in DNCB treated with dorsal skin of mice and T+I-treated HaCaT cells. However, Derma-Hc recovered the expression of FLG both in vivo and in vitro level. These results suggested that Derma-Hc facilitated keratinocyte differentiation by restoring the EDC marker.

Recent studies have illustrated that FLG involved in proteases and protease inhibitor become dysregulated in keratinocytes of AD, altering the balance between them, inversely [51]. Hyperkeratosis in epidermis is thoroughly regulated by serine proteases such as KLK5 or KLK7 and serine protease inhibitors such as SPINK5 [14]. KLK-associated peptidases, also known as stratum corneum enzyme are activated at a slightly alkaline pH beyond the normal pH range of 4.5 to 5.5 [52]. KLK5 has a tryptic activity regulating the KLK cascade in the stratum corneum which induce increases of skin permeability and inflammation [53]. In addition, KLK7 is a key chymotryptic enzyme in AD-like lesion that destroys the epidermal barrier homeostasis [53]. Conversely, SPINK5, also known as lymphoepithelial Kazal-type 5 serine protease inhibitor (LEKTI) is responsible for pH-dependent regulation in allergic manifestation [54]. Overall, the pH value of skin is increased by lowering the expression of FLG. Activation of KLK families is elevated, thereby SPINK5 is downregulated in response to pH dependency. Expressions of mRNA levels of serine protease enzymatic activities including KLK5 and KLK7 were increased, thereby the expression of SPINK5 was declined in DNCB-treated mice and human keratinocyte treated with TI, whereas Derma-Hc treatment down-regulated the gene expressions of KLK5 and KLK7. In addition, Derma-Hc restored SPINK5 level in dorsal skin and HaCaT cells. From these findings, Derma-Hc exhibited ameliorative effect against lichenification by modulating epidermal barrier homeostasis.

JAK1-STAT3 signaling is greatly involved in terminal differentiation in keratinocytes [55]. JAK-STAT signal pathway has been known to induce epidermal hyperplasia and hyperkeratosis [56]. Hyperactivation of JAK1 is followed by increasing the expressions of KLK families [57]. In addition, STAT3 regulates genes associated with EDC markers including FLG [58]. For that reason, targeting JAK1-STAT3 signal pathway could interrupt the progression of AD. JAK1 was mainly bound on receptors of IL-22 to initiate phosphorylation of JAK1. Then, phosphorylated JAK1 phosphorylates STAT3 in sequence, thereby phosphorylated STAT3 translocate into the nucleus in keratinocyte [59]. Topical application of Derma-Hc downregulated expressions of phosphorylated JAK1 and STAT3 compared to the control group. In addition, Derma-Hc treatment dose-dependently decreased the p-form of JAK1 and STAT3 in HaCaT cells. These data suggested that Derma-Hc effectively prevents transducing signals as an inhibitor of JAK1 and STAT3 pathway.

Stimulation of the JAK1-STAT3 signal pathway upon IL-22 derived from the Th22 cell is predominantly activated in chronic AD skin [60]. Conversion of the acute phase to the chronic phase in AD is demonstrated by not only activation of Th1 and Th17 cell but also Th22 cell activity [61]. In particular, Chronic AD is characterized by recruitments of Th1, Th22, and Th17 subsets, which disrupt activation of keratinocyte differentiation markers [30]. The role for IL-22 in pathological AD promoted abnormal epidermal hyper-proliferation and impaired keratinocyte differentiation, resulting in epidermal hyperplasia and skin barrier dysfunction [58,62]. Thus, the molecular level of IL-22 was evaluated in dorsal skin tissues and human keratinocytes. Derma-Hc treatment significantly reduced the expression of IL-22 compared with DNCB-induced mice and T+I-treated HaCaT cells, respectively. Our study demonstrated that Derma-Hc inhibited the main driver of chronic AD as for IL-22.

## 4. Materials and Methods

### 4.1. Derma-Hc Preparation

Derma-Hc consists of five natural herbs including *Astragalus membranaceus*, *Schizonepeta tenuifolia*, *Cryptotympana pustulata*, *Angelica sinensis*, and *Arctium lappa* purchased from Dong-Yang Herb (Seoul, Korea). Dried 20 g of each herb (total 100 g) were extracted with 2 L of distilled water at 100 °C for 2 h. The extraction was filtered through a solid suspension filtering apparatus. Then, the filtered solution was concentrated at 80 °C for 3 h to 23 mL and the concentrated solution was lyophilized by freeze-drying for 72 h. The yields of the Derma-Hc extract were 20.21%.

### 4.2. UPLC-ESI-QTOF MS/MS Analysis for Identification of Derma-Hc

Chromatographic analysis of the extract was performed to identify and provide chemical components to qualify and identify of the Derma-Hc. The liquid chromatography-mass spectrometry system consisted of a Thermo Scientific Vanquish UHPLC system (Thermo Fisher Scientific, Sunnyvale, CA, USA) with an ACQUITY UPLC HSS T3 column (2.1 mm × 100 mm, 1.8 μm; WatersTM) and a Triple TOF5600+ mass spectrometer system (QTOF MS/MS, SCIEX, Foster City, CA, USA). The QTOF MS was equipped with an electro spray ionization (ESI) source and was used to complete the high-resolution experiment. The elution program for UHPLC separation, which used 0.1% formic acid in water as eluent A and 0.1% formic acid in acetonitrile as eluent B, was as follows: 0–1 min; 5% B, 1–4 min; 5–15% B, 4–11 min; 15–35% B, 11–17 min; 35–50% B, 17–19 min; 50–100%, 19–24 min; 100% and equilibration with 5% B for 4 min at a flow rate of 0.4 mL/min. The column was at 40 °C and the auto-sampler was maintained at 4 °C. The injection volume of each sample solution was 2 μL.

We conducted a UPLC-ESI-QTOF MS/MS analysis to determine the chemical profile and to identify the constituents from extract (Table S1). The chromatogram shows representative components in the Derma-Hc (Figure S1) consisting of five herbs; AM, ST, CP, AS, and AL. We identified calycosin 7-O-β-glucoside, nodakenin, hesperidin, luteolin, formonoetin, astragaloside IV, and astragaloside I (Figure S1A) and confirmed with reference standards (Figure S1B) using LC-MS/MS in positive ion mode. The analysis of Derma-Hc using LC-MS/MS showed the presence of formononetin 7-O-β-D-glucopyranoside, arctiin, calycosin, arctigenin, astragaloside IV, astragaloside I, decursin, and decursinol angelate, identified tentatively. In addition, the chemical profile using high-resolution mass spectrometry found an unknown compound (peak # 10) and provided the chemical formula and fragmentation ions in ms/ms spectra.

### 4.3. Animal Treatment

Five-week-old male BALB/c were purchased from DBL (Eumseong, Korea). Mice were housed at the animal center of Kyung Hee University (Seoul, Korea) in a temperature-controlled room (23 ± 1 °C) with relative humidity (50% ± 10%) and maintained 12 h light/dark cycle. All procedures involving animals were approved in accordance with the regulations of the Guide for the Care and Use of Laboratory Animals of Kyung Hee University (KHUASP(SE)-20-053). After one-week acclimation, mice were randomly divided into four groups (*n* = 5). The normal control group was sensitized and applied with normal saline (NOR). The negative control group was sensitized with 2,4-dinitrochlorobenzene (DNCB). The positive control group was sensitized with DNCB and topically treated with 10 μM dexamethasone (DEX). The Derma-Hc group was sensitized with DNCB and applied with 20 mg/mL of Derma-Hc dissolved in distilled water. One day after dorsal hair was shaved off using an electric razor, 200 μL of 1% DNCB dissolved in 4:1 ratio of acetone:olive oil was topically applied to the dorsal skin at days 1, 2, and 3 for induction of AD-like symptoms. Next, for the second induction, 200 μL of 4% sodium dodecyl sulfate (SDS) was treated 4 h before Derma-Hc was topically administered. After 4 h later, 200 μL of 0.5% DNCB was administered to the dorsal skin on days 7 to 20. Following the day, the mice were anesthetized with 1% avertin and blood samples were collected by cardiac puncture. The dorsal skins from the mice were dissected and stored after sacrifice.

### 4.4. Evaluation of Dermatitis Severity

Symptoms of AD including erythema/hemorrhage, scarring/dryness, edema, and excoriation/erosion on dorsal skin were evaluated as follows: 0 (none), 1 (mild, <20%), 2 (moderate, 20–60%), 3 (severe >60%) on days 4, 7, 14, and 21. The sum of the four individual scores was defined as the dermatitis severity score. A single researcher blindly assessed the severity of AD-like symptoms. Mice were anesthetized with 1% avertin and photographed by digital camera on the final day of the experiment.

### 4.5. Measurement of Scratching Behavior Frequency

The day before the end of the experiment, the mice were adapted for at least 10 min in the observation chamber prior to observing scratching. After 30 min, 200 μL of 0.5% DNCB administration, the scratching behavior was recorded with a digital camera for 20 min. The frequency of scratching was counted using double-blind assessment. Scratching behavior was defined as the movement for scratching the back with hind paws.

### 4.6. Histopathological Examination

At the end of the experiment, the dorsal skin of each mouse was collected, excised and fixed with 4% neutral-buffered formalin and embedded in paraffin. Dorsal cutaneous tissues were sliced to 5 μm of thickness and deparaffinized section were stained with hematoxylin and eosin (H&E) to evaluate the thickness of epidermis and dermis. The thickness of epidermis and dermis was quantified using Image J program (ver. 1.38e; National Institutes of Health, Bethesda, MD, USA). We also investigated the number of mast cells by staining with toluidine blue. Mast cells were counted in five sites randomly designated sites at 100× magnification.

### 4.7. Immunofluorescence Analysis

The dorsal skin from each mouse was fixed with 4% neutral-buffered formalin. The dorsal skin tissues were deparaffinized with xylene and hydrated with gradual ethanol. The skin tissues were incubated with primary antibody (anti-DSC1, # sc-398590, Santa Cruz, CA, USA) overnight at 4 °C. The next day, the sections were incubated with a secondary antibody, Alexa Fluor 594 goat Anti-mouse IgG (Invitrogen, Carlsbad, CA, USA). TO-PRO-3 (Invitrogen, Carlsbad, CA, USA) was used for the nuclei. Confocal microscopy was carried out on Zeiss LSM5 pascal vario Two RGB.

### 4.8. Cell Culture

Human keratinocyte HaCaT cells were maintained in Dulbecco’s modified Eagle’s medium (DMEM) containing 1% of penicillin (1 × 105 U/L), streptomycin (100 mg/mL), and 10% of fetal bovine serum (FBS) at 37 °C in a humidified CO2 (5%) incubator. HaCaT cells were seeded at a density of 5 × 10^5 cells/well. Then, HaCaT cells were treated with gradual concentration of Derma-Hc (1, 10, and 100 μg/mL) in presence of TNF-α (10 ng/mL) and IFN-γ (10 ng/mL) (TI) for 24 h in serum-free media. The next day, cells were harvested.

### 4.9. Western Blot Analysis

Total protein of dorsal skin from mice and HaCaT keratinocytes were extracted using 1 × radioimmunoprecipitation assay (RIPA, T&I, Chuncheon, Korea) buffer supplemented with protease inhibitor cocktail tablet (Roche, Penzberg, Germany). Lysates were centrifuged for 20 min at 4 °C and the supernatants were collected. Western blot assay was performed using Bradford methods. Lysates of 15 μg of protein were electrophoresed using 7.5% SDS-polyacrylamide gel at 80 V for 2 h. Sample proteins were electrophoretically transferred onto a polyvinylidene fluoride (PVDF) membrane. After the membrane was blocked with 5% BSA in Tris-buffered saline containing 0.05% Tween-20 (TBS-T) for 2 h. The membranes were incubated with primary antibodies specific for β-actin (1:1000), JAK1 (1:1000), phospho (p)-JAK1 (1:1000) from cell signaling technology, STAT3 (1:1000), and phospho-STAT3 (1:1000) from Santa Cruz biotechnology overnight at 4 °C. After three times of washing with TBS-T, the membrane was incubated with horseradish peroxidase-conjugated secondary antibodies for 1 h at RT. The blots were visualized using enhanced chemiluminescence (ECL) by chemiluminescence imaging system (Young In, Seoul, Korea).

### 4.10. Reverse Transcription-Polymerase Chain Reaction (RT-PCR) Analysis

Total RNA was isolated from dorsal skin tissues and HaCaT cells using Trizol reagent. One μg of total RNA was reverse transcribed using Maxime RT PreMix kit (iNtRON Biotechnology, Inc., Seongnam, Korea). PCR was conducted using a mixture of cDNA, 5 pmol of forward and reversed primer and RNase free water. The amplification condition was as follow: pre-denaturation at 94 °C for 10 min, followed by denaturation at 94 °C, annealing at 60 °C for 30 s and extension at 72 °C, 1 min. The amplified PCR products were analyzed on 2% agarose gel electrophoresis. The primers used in this study are listed in Table 1.

### 4.11. Statistical Analysis

The data are presented as mean ± standard error of the mean (S.E.M.). One-way analysis of variance (ANOVA) using the Tukey post-hoc test was used to identify significant differences. A *p*-value < 0.05 was considered significant between the individual groups. Significance was calculated using GraphPad Prism 5 (GraphPad Software Inc., San Diego, CA, USA).

## 5. Conclusions

In conclusion, Derma-Hc inhibited the development of lichenification including AD-like phenotypes such as pruritus, epidermal hyperplasia, and hyperkeratosis. In addition, integrity of epidermis is recovered by up-regulating the expression of DSC1. External application of Derma-Hc modulated epidermal differentiation markers including FLG, KLK5, KLK7, and SPINK5 via suppressing the IL-22-mediated JAK1-STAT3 signal pathway in DNCB-induced dorsal skin and TI-treated human keratinocytes. Derma-Hc might be a potential therapeutic treatment to prevent lichenification of atopic dermatitis.

## Figures and Tables

**Table 1:** Sequence of reverse transcription PCR primers.

Gene	Forward Primer (5′→3′)	Reverse Primer (5′→3′)
*IL-22*	TGAGTGAGCGCTGCTATCTG	TGTGCTTAGCCTGTTGCTGA
*FLG*	TGAAGCCTATGACACCACTGA	TCCCCTACGCTTTCTTGTCCT
*KLK5*	GCCACACTGCAGGAAGAAA	GGATTTGACCCCCTGGAA
*KLK7*	GCATCCCCGACTCCAAGAA	CAGGGTACCTCTGCACACCAA
*SPINK5*	GATCCTATTGAGGGTCTAGAT	ATTACCATGTGTCTTGCCATC
*GAPDH*	GGCATGGACTGTGGTCATGA	TTCACCACCATGGAGAAGGC

## Data Availability

The data presented in this study are available on request from the corresponding author.

## Supplementary Materials

The following are available online at https://www.mdpi.com/1422-0067/22/5/2359/s1, Figure S1: The representative Base Peak Chromatogram (BPC) of the Derma-Hc extract (A) and the extracted ion chromatogram (XIC) of the six reference standards (B) were obtained using UPLC-ESI-QTOF MS/MS analysis in positive ion mode. Table S1: Identification of chemical components authentically or tentatively in the Derma-Hc using LC-ESI-QTOF MS/MS.

## Abbreviations

AD	Atopic dermatitis
AL	*Arctium lappa*
AM	*Astragalus membranaceus*
AS	*Angelica sinensis*
CP	*Cryptotympana pustulata*
DEX	Dexamethasone
DNCB	2,4-Dinitrochlorobenzene
FLG	Filaggrin
IFN-γ	Interferon-gamma
Ig	Immunoglobulin
IL	Interleukin
JAK1	Janus kinase 1
KLK5	Kallikrein-related peptidase 5
KLK7	Kallikrein-related peptidase 7
QTOF	Quadrupole time-of-flight
RT	Retention time
*SPINK5*	Serine protease inhibitor Kazal-type 5
ST	*Schizonepeta tenuifolia*
STAT3	Signal transducer and activator of transcription 3
Th	T helper
TNF-α	Tumor necrosis factor-alpha

## References

[1] Jeon J.H., Kwon S.C., Park D., Shin S., Jeong J.H., Park S.Y., Hwang S.Y., Kim Y.B., Joo S.S. (2009). Anti-allergic effects of white rose petal extract and anti-atopic properties of its hexane fraction. Arch. Pharm. Res..

[2] Nettis E., Ortoncelli M., Pellacani G., Foti C., Di Leo E., Patruno C., Rongioletti F., Argenziano G., Ferrucci S.M., Macchia L. (2020). A Multicenter Study on the Prevalence of Clinical Patterns and Clinical Phenotypes in Adult Atopic Dermatitis. J. Investig. Allergol. Clin. Immunol..

[3] Lee J.H., Ki H.H., Kim D.K., Lee Y.M. (2018). Triticum aestivum sprout extract attenuates 2,4dinitrochlorobenzeneinduced atopic dermatitislike skin lesions in mice and the expression of chemokines in human keratinocytes. Mol. Med. Rep..

[4] De Benedetto A., Agnihothri R., McGirt L.Y., Bankova L.G., Beck L.A. (2009). Atopic dermatitis: A disease caused by innate immune defects?. J. Investig. Dermatol..

[5] Bylund S., von Kobyletzki L.B., Svalstedt M., Svensson A. (2020). Prevalence and Incidence of Atopic Dermatitis: A Systematic Review. Acta Derm. Venereol..

[6] Yarbrough K.B., Neuhaus K.J., Simpson E.L. (2013). The effects of treatment on itch in atopic dermatitis. Dermatol. Ther..

[7] Carter K.F., Dufour L.T., Ballard C.N. (2004). Identifying secondary skin lesions. Nursing.

[8] Lou H., Lu J., Choi E.B., Oh M.H., Jeong M., Barmettler S., Zhu Z., Zheng T. (2017). Expression of IL-22 in the Skin Causes Th2-Biased Immunity, Epidermal Barrier Dysfunction, and Pruritus via Stimulating Epithelial Th2 Cytokines and the GRP Pathway. J. Immunol..

[9] Eyerich K., Eyerich S. (2015). Th22 cells in allergic disease. Allergo J. Int..

[10] Kawakami T., Ando T., Kimura M., Wilson B.S., Kawakami Y. (2009). Mast cells in atopic dermatitis. Curr. Opin. Immunol..

[11] Amin K. (2012). The role of mast cells in allergic inflammation. Respir. Med..

[12] Wang A.X., Xu Landen N. (2015). New insights into T cells and their signature cytokines in atopic dermatitis. IUBMB Life.

[13] Fujita H. (2013). The role of IL-22 and Th22 cells in human skin diseases. J. Dermatol. Sci..

[14] Rerknimitr P., Otsuka A., Nakashima C., Kabashima K. (2017). The etiopathogenesis of atopic dermatitis: Barrier disruption, immunological derangement, and pruritus. Inflamm. Regen..

[15] Caubet C., Jonca N., Brattsand M., Guerrin M., Bernard D., Schmidt R., Egelrud T., Simon M., Serre G. (2004). Degradation of corneodesmosome proteins by two serine proteases of the kallikrein family, SCTE/KLK5/hK5 and SCCE/KLK7/hK7. J. Invest. Dermatol..

[16] Simpson E.L. (2010). Atopic dermatitis: A review of topical treatment options. Curr. Med. Res. Opin..

[17] Tidwell W.J., Fowler J.F. (2018). T-cell inhibitors for atopic dermatitis. J. Am. Acad. Dermatol..

[18] Thomsen S.F. (2014). Atopic dermatitis: Natural history, diagnosis, and treatment. ISRN Allergy.

[19] Dattola A., Bennardo L., Silvestri M., Nistico S.P. (2019). What’s new in the treatment of atopic dermatitis?. Dermatol. Ther..

[20] Mostow E. (2019). A systematic review of the safety and efficacy of systemic corticosteroids in atopic dermatitis. J. Am. Acad. Dermatol..

[21] Thaci D., Salgo R. (2010). Malignancy concerns of topical calcineurin inhibitors for atopic dermatitis: Facts and controversies. Clin. Dermatol..

[22] Jeon Y.C. (2016). Treatment for an Adult Patient with Psoriasis with Traditional Korean Medicine, Especially Sa-Am Acupuncture and Herbal Medicine. J. Acupunct. Meridian Stud..

[23] Jo S.Y., Kim M.H., Lee H., Lee S.H., Yang W.M. (2020). Ameliorative and Synergic Effects of Derma-H, a New Herbal Formula, on Allergic Contact Dermatitis. Front. Pharmacol..

[24] Kim J.H., Kim M.H., Yang G., Huh Y., Kim S.H., Yang W.M. (2013). Effects of topical application of Astragalus membranaceus on allergic dermatitis. Immunopharmacol. Immunotoxicol..

[25] Choi Y.Y., Kim M.H., Kim J.H., Jung H.S., Sohn Y., Choi Y.J., Hwang M.K., Kim S.H., Kim J., Yang W.M. (2013). Schizonepeta tenuifolia inhibits the development of atopic dermatitis in mice. Phytother. Res..

[26] Chen H.Y., Lin Y.H., Huang J.W., Chen Y.C. (2015). Chinese herbal medicine network and core treatments for allergic skin diseases: Implications from a nationwide database. J. Ethnopharmacol..

[27] Lee J., Choi Y.Y., Kim M.H., Han J.M., Lee J.E., Kim E.H., Hong J., Kim J., Yang W.M. (2016). Topical Application of Angelica sinensis Improves Pruritus and Skin Inflammation in Mice with Atopic Dermatitis-Like Symptoms. J. Med. Food.

[28] Sohn E.H., Jang S.A., Joo H., Park S., Kang S.C., Lee C.H., Kim S.Y. (2011). Anti-allergic and anti-inflammatory effects of butanol extract from *Arctium lappa* L.. Clin. Mol. Allergy.

[29] Petersen T.K. (2006). In vivo pharmacological disease models for psoriasis and atopic dermatitis in drug discovery. Basic Clin. Pharmacol. Toxicol..

[30] Kim J., Kim B.E., Leung D.Y.M. (2019). Pathophysiology of atopic dermatitis: Clinical implications. Allergy Asthma Proc..

[31] Sharma L. (2001). Diagnostic clinical features of atopic dermatitis. Indian J. Dermatol. Venereol. Leprol..

[32] Vender R.B. (2002). Alternative treatments for atopic dermatitis: A selected review. Skin Ther. Lett..

[33] Zhu Y., Yu X., Ge Q., Li J., Wang D., Wei Y., Ouyang Z. (2020). Antioxidant and anti-aging activities of polysaccharides from Cordyceps cicadae. Int. J. Biol. Macromol..

[34] Chan Y.S., Cheng L.N., Wu J.H., Chan E., Kwan Y.W., Lee S.M., Leung G.P., Yu P.H., Chan S.W. (2011). A review of the pharmacological effects of Arctium lappa (burdock). Inflammopharmacology.

[35] Hanifin J.M., Thurston M., Omoto M., Cherill R., Tofte S.J., Graeber M. (2001). The eczema area and severity index (EASI): Assessment of reliability in atopic dermatitis. EASI Evaluator Group. Exp. Dermatol..

[36] Ikoma A., Steinhoff M., Stander S., Yosipovitch G., Schmelz M. (2006). The neurobiology of itch. Nat. Rev. Neurosci..

[37] Rinaldi G. (2019). The Itch-Scratch Cycle: A Review of the Mechanisms. Dermatol. Pract. Concept..

[38] Arima K., Ohta S., Takagi A., Shiraishi H., Masuoka M., Ontsuka K., Suto H., Suzuki S., Yamamoto K., Ogawa M. (2015). Periostin contributes to epidermal hyperplasia in psoriasis common to atopic dermatitis. Allergol. Int..

[39] Matsunaga Y., Ogura Y., Ehama R., Amano S., Nishiyama T., Tagami H. (2007). Establishment of a mouse skin model of the lichenification in human chronic eczematous dermatitis. Br. J. Dermatol..

[40] Nakahigashi K., Kabashima K., Ikoma A., Verkman A.S., Miyachi Y., Hara-Chikuma M. (2011). Upregulation of aquaporin-3 is involved in keratinocyte proliferation and epidermal hyperplasia. J. Investig. Dermatol..

[41] Gu H., Kim W.H., An H.J., Kim J.Y., Gwon M.G., Han S.M., Leem J., Park K.K. (2018). Therapeutic effects of bee venom on experimental atopic dermatitis. Mol. Med. Rep..

[42] Irani A.M., Sampson H.A., Schwartz L.B. (1989). Mast cells in atopic dermatitis. Allergy.

[43] Sehra S., Serezani A.P.M., Ocana J.A., Travers J.B., Kaplan M.H. (2016). Mast Cells Regulate Epidermal Barrier Function and the Development of Allergic Skin Inflammation. J. Investig. Dermatol..

[44] Donetti E., Bedoni M., Boschini E., Dellavia C., Barajon I., Gagliano N. (2005). Desmocollin 1 and desmoglein 1 expression in human epidermis and keratinizing oral mucosa: A comparative immunohistochemical and molecular study. Arch. Dermatol. Res..

[45] Totsuka A., Omori-Miyake M., Kawashima M., Yagi J., Tsunemi Y. (2017). Expression of keratin 1, keratin 10, desmoglein 1 and desmocollin 1 in the epidermis: Possible downregulation by interleukin-4 and interleukin-13 in atopic dermatitis. Eur. J. Dermatol..

[46] Matsui T., Amagai M. (2015). Dissecting the formation, structure and barrier function of the stratum corneum. Int. Immunol..

[47] Sandilands A., Sutherland C., Irvine A.D., McLean W.H. (2009). Filaggrin in the frontline: Role in skin barrier function and disease. J. Cell Sci..

[48] O’Regan G.M., Sandilands A., McLean W.H., Irvine A.D. (2009). Filaggrin in atopic dermatitis. J. Allergy Clin. Immunol..

[49] Brown S.J., McLean W.H. (2012). One remarkable molecule: Filaggrin. J. Investig. Dermatol..

[50] Scharschmidt T.C., Man M.Q., Hatano Y., Crumrine D., Gunathilake R., Sundberg J.P., Silva K.A., Mauro T.M., Hupe M., Cho S. (2009). Filaggrin deficiency confers a paracellular barrier abnormality that reduces inflammatory thresholds to irritants and haptens. J. Allergy Clin. Immunol..

[51] Lee H.J., Lee S.H. (2014). Epidermal permeability barrier defects and barrier repair therapy in atopic dermatitis. Allergy Asthma Immunol. Res..

[52] Ekholm I.E., Brattsand M., Egelrud T. (2000). Stratum corneum tryptic enzyme in normal epidermis: A missing link in the desquamation process?. J. Investig. Dermatol..

[53] Morizane S. (2019). The Role of Kallikrein-Related Peptidases in Atopic Dermatitis. Acta Med. Okayama.

[54] Egawa G., Kabashima K. (2016). Multifactorial skin barrier deficiency and atopic dermatitis: Essential topics to prevent the atopic march. J. Allergy Clin. Immunol..

[55] Fridman J.S., Scherle P.A., Collins R., Burn T., Neilan C.L., Hertel D., Contel N., Haley P., Thomas B., Shi J. (2011). Preclinical evaluation of local JAK1 and JAK2 inhibition in cutaneous inflammation. J. Investig. Dermatol..

[56] He H., Guttman-Yassky E. (2019). JAK Inhibitors for Atopic Dermatitis: An Update. Am. J. Clin. Dermatol..

[57] Yasuda T., Fukada T., Nishida K., Nakayama M., Matsuda M., Miura I., Dainichi T., Fukuda S., Kabashima K., Nakaoka S. (2016). Hyperactivation of JAK1 tyrosine kinase induces stepwise, progressive pruritic dermatitis. J. Clin. Investig..

[58] Brunner P.M., Pavel A.B., Khattri S., Leonard A., Malik K., Rose S., Jim On S., Vekaria A.S., Traidl-Hoffmann C., Singer G.K. (2019). Baseline IL-22 expression in patients with atopic dermatitis stratifies tissue responses to fezakinumab. J. Allergy Clin. Immunol..

[59] Welsch K., Holstein J., Laurence A., Ghoreschi K. (2017). Targeting JAK/STAT signalling in inflammatory skin diseases with small molecule inhibitors. Eur. J. Immunol..

[60] Lejeune D., Dumoutier L., Constantinescu S., Kruijer W., Schuringa J.J., Renauld J.C. (2002). Interleukin-22 (IL-22) activates the JAK/STAT, ERK, JNK, and p38 MAP kinase pathways in a rat hepatoma cell line. Pathways that are shared with and distinct from IL-10. J. Biol. Chem..

[61] Bernard F.X., Morel F., Camus M., Pedretti N., Barrault C., Garnier J., Lecron J.C. (2012). Keratinocytes under Fire of Proinflammatory Cytokines: Bona Fide Innate Immune Cells Involved in the Physiopathology of Chronic Atopic Dermatitis and Psoriasis. J. Allergy.

[62] Hanel K.H., Cornelissen C., Luscher B., Baron J.M. (2013). Cytokines and the skin barrier. Int. J. Mol. Sci..

