# Supplementary material for: Derma-Hc, a New Developed Herbal Formula, Ameliorates Cutaneous Lichenification in Atopic Dermatitis
Source: Int J Mol Sci. 2021 Feb 26;22(5):2359. doi: 10.3390/ijms22052359 (PMC7956340; doi:10.3390/ijms22052359)
Supplement: Supplementary file 1 [file ijms-22-02359-s001.pdf]

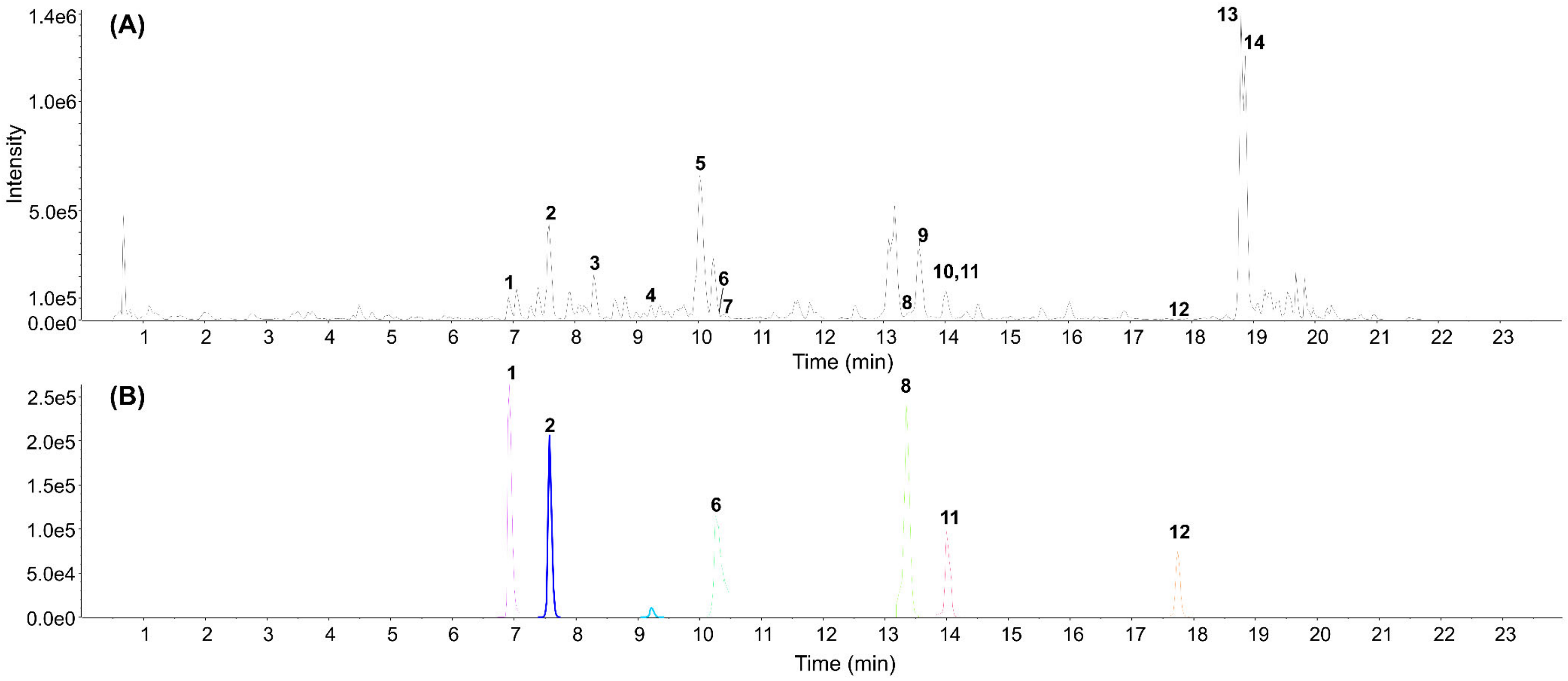

Figure 1. The representative Base Peak Chromatogram (BPC) of the Derma-Hc extract (**A**) and the extracted ion chromatogram (XIC) of the six reference standards (**B**) were obtained using UPLC-ESI-QTOF MS/MS analysis in positive ion mode.

Table S1. Identification of chemical components authentically or tentatively in the Derma-Hc extract using LC-ESI-QTOF MS/MS

No.	Mass (Da)	Found at RT (min)	Adduct	Found at Mass (Da)	Error (ppm)	MS/MS Product Ions	Name	Formula	Indetified with	Originated Plant
**1**	446.1213	6.93	$[M+H]^+$	447.1286	0.4	285.0769, 270.0538, 253.0508, 225.0555, 213.0554, 137.0238	calycosin 7-O-β-glucoside	$C_{22}H_{22}O_{10}$	†	*A. membrancaceus*
**2**	408.1420	7.58	$[M+H]^+$	409.1493	−0.7	247.0965, 229.0862, 187.0391, 175.0391, 201.0911	nodakenin	$C_{20}H_{24}O_9$	†	*A. sinensis*
**3**	610.1898	8.31	$[M+H]^+$	611.1971	−1.6	303.0861, 263.0051, 195.0291, 177.0548, 153.0186	hesperidin	$C_{28}H_{34}O_{15}$	†	*S. tenuifolia*
**4**	430.1264	9.23	$[M+H]^+$	431.1337	0.7	269.0808, 254.0575, 237.0575, 213.0914	formononetin 7-O-β-D-glucopyranoside	$C_{22}H_{22}O_9$	*	*A. membrancaceus*
**5**	534.2101	10.04	$[M+H]^+$	535.2174	−1.0	373.1648, 355.1534, 305.1170, 137.0598	arctiin	$C_{27}H_{34}O_{11}$	#	*A. lappa*
**6**	286.0477	10.29	$[M+H]^+$	287.0550	0.8	287.0551, 241.0483, 153.0175, 135.0433	luteolin	$C_{15}H_{10}O_6$	†	*S. tenuifolia*
**7**	284.0685	10.39	$[M+H]^+$	285.0758	2.2	270.0529, 225.0544, 213.0549, 137.0237	calycosin	$C_{16}H_{12}O_5$	#	*A. membrancaceus*
**8**	268.0736	13.36	$[M+H]^+$	269.0808	−0.4	253.0500, 237.0549, 226.0627, 197.0597, 181.0636	formononetin	$C_{16}H_{12}O_4$	†	*A. membrancaceus*
**9**	372.1573	13.58	$[M+H]^+$	373.1646	1.6	305.1181, 177.0907, 151.0755, 137.0595, 122.0366	arctigenin	$C_{21}H_{24}O_6$	#	*A. lappa*
**10**	731.2942	14.01	$[M+H]^+$	732.3015	−0.7	655.2539, 561.2122, 531.2007, 137.0596	unknown	$C_{40}H_{45}NO_{12}$	*	
**11**	784.4609	14.01	$[M+H]^+$	785.4682	0.2	473.3624, 437.3425, 143.1066, 125.0964	astragaloside IV	$C_{41}H_{68}O_{14}$	†	*A. membrancaceus*
**12**	868.4820	17.74	$[M+H]^+$	869.4893	0.8	671.4188, 419.3275, 217.0711, 157.0481	astragaloside I	$C_{45}H_{72}O_{16}$	†	*A. membrancaceus*
**13**	328.1311	18.83	$[M+H]^+$	329.1384	0.2	329.1395, 247.0973, 229.0862, 213.0549	decursin	$C_{19}H_{20}O_5$	#	*A. sinensis*
**14**	328.1311	18.83	$[M+H]^+$	329.1384	0.2	329.1395, 247.0973, 229.0862, 213.0549	decursinol angelate	$C_{19}H_{20}O_5$	#	*A. sinensis*

In-house ms/ms library and online database; such as GNPS, MASS bank or Metlin; † Reference standard; * Exact mass, Isotope pattern, and/or ms/ms spectra with previous report
